# Sporadic implementation of UK familial mammographic surveillance guidelines 15 years after original publication

**DOI:** 10.1038/s41416-019-0631-2

**Published:** 2019-11-25

**Authors:** D. Gareth Evans, Maria Edwards, Stephen W. Duffy, Cancer Genetics Group clinical leads, Julian Adlard, Munaza Ahmed, Julian Barwell, Angela Brady, Paul Brennan, Carole Brewer, Jacqueline Cook, Tabib Dabir, Rosemarie Davidson, Alan Donaldson, Angela George, David Goudie, Lynn Greenhalgh, Dorothy Halliday, Helen Hanson, Rachel Harrison, Anju Kulkarni, Fiona Lalloo, Kai ren Ong, Zosia Miedzybrodzka, Alex Murray, Mary Porteous, Lucy Side, Katie Snape, Marc Tischkowitz

**Affiliations:** 1 Prevent Breast Cancer Centre, Wythenshawe Hospital Manchester Universities Foundation Trust, Wythenshawe, Manchester M23 9LT UK; 2 Genomic Medicine, Division of Evolution and Genomic Sciences, The University of Manchester, Manchester Academic Health Science Centre, Manchester Universities Foundation Trust, St. Mary’s Hospital, Oxford Road, Manchester, M13 9WL UK; 3 NIHR Biomedical Research Centre (Cancer PED theme), Manchester Cancer Research Centre, University of Manchester, Christie Hospital, Withington, Manchester M20 4BX UK; 4 Cancer Research UK Centre for Epidemiology, Mathematics and Statistics, Wolfson Institute of Preventive Medicine’ Charterhouse Square, London, EC1M 6BQ UK; 5 Department of Medical Genetics, University of Cambridge and NIHR Cambridge Biomedical Research Centre, and Cancer Research UK Cambridge Centre, Cambridge Biomedical Campus, Cambridge, CB2 0QQ UK

**Keywords:** Health policy, Health services

## Abstract

The National Institute of health and Care Excellence issued guidelines on familial breast cancer screening in 2004. Such guidelines should be uniformly implemented to ensure that members of the same family with the same level of risk, but living in different areas, have the same access to screening. We assessed uptake by creating a short, six question online survey designed to assess compliance in each regional area. We used this to conduct a survey of all 22 regional genetics services. There was a 100% response to the survey allowing a complete map to be created. The devolved nations had near complete compliance with the sole exception of SW Scotland, but in England the picture was fragmented with regions representing a combined population of 26.6 million (48%) not implementing the full NICE recommendations. Fifteen years after the publication of the original guidelines, major inequity in provision for screening still occurs and a postcode lottery exists for the management of women from families with a history of breast cancer. We estimate that up to 73 preventable breast cancer deaths occur each year due to the current inequity of access. It may be time to consider alternative funding and implementation models to ensure consistent access across the country.

## Background

Guidelines are an important mechanism to ensure equitable and evidence-based delivery of medical care. In the UK (England & Wales) the National Institute of health and Care Excellence (NICE) was created 20 years ago with a strapline of ‘Improving health and social care through evidence-based guidance’. When the term ‘offer’ is utilised this infers that health commissioners should implement the recommendation as it has usually passed the test of being cost effective. Although guidelines are just ‘guidance’, the purpose of NICE guidelines was to avoid postcode lotteries where an individual in one part of a country was able to access an aspect of healthcare, whereas in another part they would be refused access. While some have intimated that that individual guidance could be ignored if it was not in the patient’s interest to follow it,^1^ even if it was NICE guidance, this is not the same as health commissioners deciding whether or not to provide the financial requirements to deliver guidance for large groups of people.

Breast screening is an important element of what can be offered to women at increased risk of breast cancer by virtue of their family history. Targeting such screening at those at higher risk reduces the negative elements of screening such as concerns regarding what is often termed ‘overdiagnosis’. Women who have seen a mother or sister die from breast cancer before 50 years of age will be concerned about having to wait until age 50, the usual starting age for national programmes to undergo mammography screening.^2^ Many countries have produced guidelines including frequently updated guidance in the USA,^3^ Netherlands^4^ and France.^5^ The first NICE guideline group for familial breast cancer was constituted in 2002 and produced its first report in May 2004.^6^ This recommended that women at moderate (lifetime risk 17–29%; 10-year risk aged 40 of ≥3%) and high risk (lifetime risk ≥30%; 10-year risk aged 40 of ≥8%) should be ‘offered’ annual mammography screening aged 40–49 years of age. It was acknowledged that a national study (FH01) was in progress and would inform a future update.^7,8^ A short update in 2006,^6^ recommended MRI screening for women at very high risk including carriers of pathogenic variants in *BRCA1, BRCA2* and *TP53* aged 30–49 years based at least in part on the UK MARIBS study results.^9^ The 2013 update^6^ was able to include the results of the FH01 mammography screening trial in women aged 40–49 and strengthen the recommendation to offer annual mammography aged 40–49 for moderate-risk and 40–59 for high-risk women. The FH01 study^8^ showed a projected mortality advantage for annual mammography surveillance compared to age matched women with no screening based on tumour characteristics at diagnosis. Approximately 3% of women aged 40 years are at moderate or high-risk of breast cancer based on family history alone,^9^ and at least half of these come forward with concerns.^10^

Some women at moderate risk were still able to access mammographic screening aged 40–49 years at least through the FH01 trial^8^ that included 76 centres across the UK, but anecdotal evidence between 2006–2011 pointed to patchy implementation of MRI screening. As a result, the National Health Service Breast Screening Programme (NHSBSP) was asked to deliver the MRI aspects of NICE guidance. In January 2012 the Advisory Committee on Breast Cancer Screening agreed to implement this and there is now equitable provision of MRI screening (aged 30–49) and annual mammography screening from 40-69 years in *BRCA1*, *BRCA2* and *TP53* carriers (no mammography for *TP53*) in England.^11^ The protocol for MRI screening included those at ‘equivalent’ high risk to a *BRCA1/BRCA2* mutation carrier. This was misinterpreted by a number of clinics to include all those at high-risk of breast cancer. In reality only about 8–10% of high-risk women qualify for the NHSBSP higher risk programme^10,12^ and the protocols have now been rewritten to clarify this. However, this uncertainty has led to many family history clinics (FHCs) not being commissioned to deliver the NICE defined high-risk annual screening to age 59 years.

These concerns over the reduction in availability of NICE recommended mammography screening led us to survey all UK genetics centres delivering cancer genetics services.

## Methods

A short, six question online survey was designed (supplementary table) to assess compliance in each regional area. The invitation was sent out on 14 March 2019 to all cancer genetics leads from the Cancer Genetics Group (http://www.ukcgg.org/) covering 22 regional centres.

## Results

**Fig. 1:**
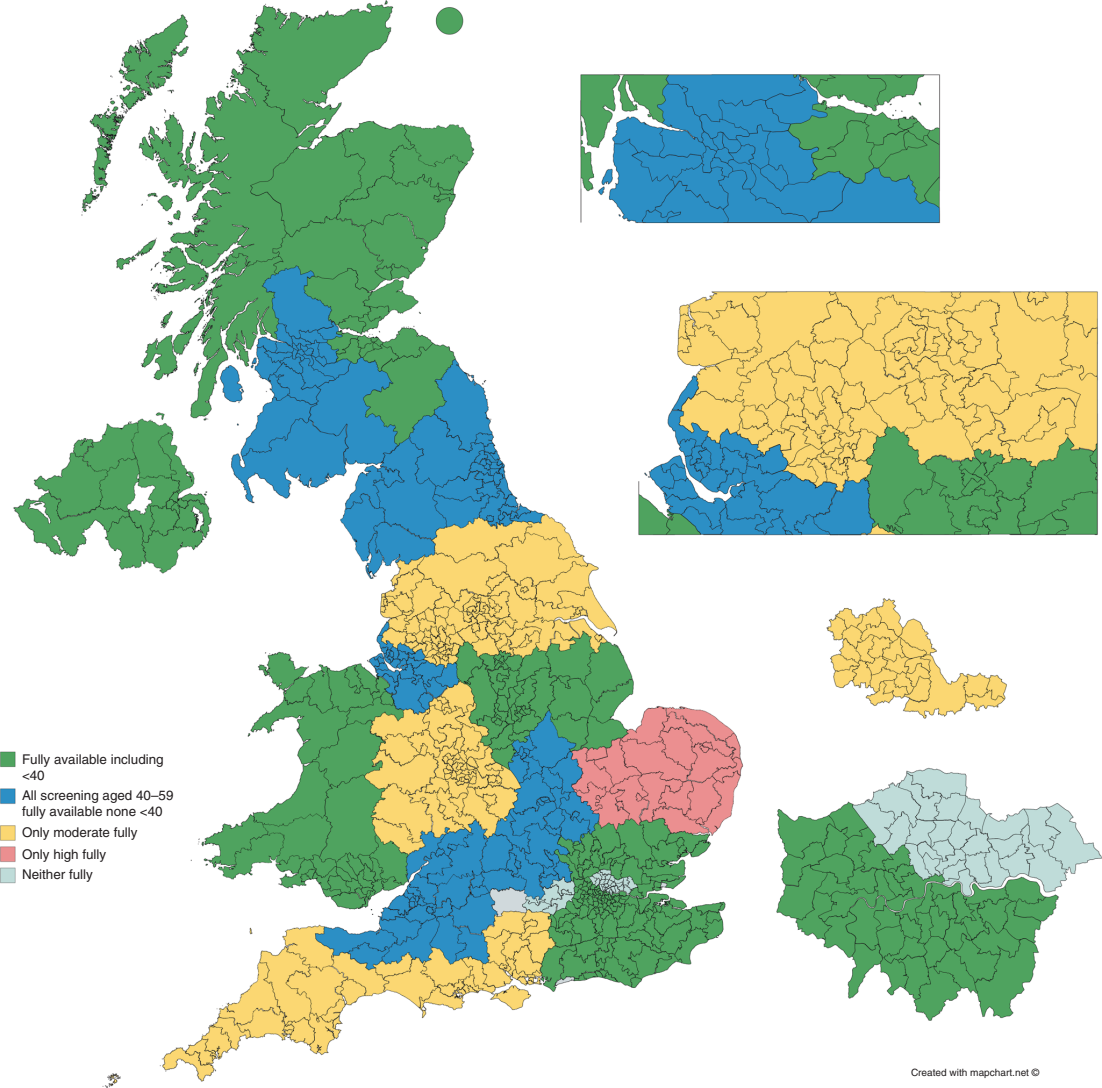
Map of mammographic screening provision by regional genetics centre. CCGs within each region are not shown, nor is the variation in provision by CCGs within a region. It is therefore possible that a CCG within a “non-compliant” region may be fully compliant and vice versa.

There was a 100% response to the survey allowing a complete map to be created. The devolved nations of Wales, Northern Ireland and Scotland had near complete compliance with the NICE recommended mammography screening ‘offer’ with the sole exception of parts of SW Scotland that only screened moderate-risk women 2-yearly (Fig. 1; Table 1). In England the picture was far patchier with regions representing a combined total population of 26.6 million (48% of the total population) not supplying all NICE recommended mammography screening. Regions covering 10.4 million people did not have coverage for Moderate-risk screening and in some areas moderate-risk screening was only available to those carrying pathogenic variants in “moderate risk” genes such as *ATM* or *CHEK2*, which represent only a tiny proportion of all those potentially eligible.

**Table 1:** Provision of NICE approved mammography screening for familial breast cancer.

Mammography screening provision	Fully available including <40	All screening aged 40–59 fully available none <40	Only moderate fully	Only high fully	neither fully	Total Population
	South East Scotland	North West Thames	Peninsula	East Anglia	South West Thames	
	South Yorkshire, North Derbyshire, North Notts	Thames Valley	Wessex		London NE	
	South East London, Kent, East Sussex	Leicester, Leicestershire and Rutland	West Midlands and surrounding counties			
	Northern Ireland	North East and North Cumbria	Yorkshire and Humber			
	North of Scotland	Cheshire & Merseyside	Manchester/ North West England			
	Tayside	Bristol and Somerset				
	Wales	West of Scotland				
Population	14,550,000	19,200,000	19,200,000	3,000,000	7,400,000	63,350,000

## Discussion

NICE guidelines are considered as providing high-level evidence and are often utilised around the world.^13^ We have shown that 15 years after the original NICE guidance to offer annual mammography screening to women at moderate-risk aged 40–49 there is still patchy provision across England in particular. Based on the results we can attempt to estimate the possible impact of the observed discrepancies in screening. From Table 1, 10.4 million individuals (7.4 million + 3 million) live in areas not receiving the recommended surveillance for moderate risk. Thus, we estimate that up to 16.4% of eligible women (10.4/63.35 million) are not receiving the appropriate moderate-risk surveillance. Of the 4.3 million women aged 40–49 in the UK, it is estimated that 8% have ten-year risk in excess of 3% at age 40, that is 344,000 women.^9^ From the above, up to 16.4% of these are not receiving the recommended annual mammography surveillance, i.e. 56,416 women. In the FH01 study, it was estimated that 7.8 breast cancer deaths are prevented in this population per 10,000 surveillance episodes.^14^ Thus, up to 44 breast cancer deaths year would be prevented if the guidelines were fully adhered to.

The provision for high-risk screening in women aged 40–59 is even worse meaning that women at higher levels of breast cancer risk in their 40’s are unable to access screening. This is despite strengthening in the evidence base in the 2006 and 2013 updates to the familial breast cancer guidance.^6^ From Table 1, it is estimated that up to 42% of the population (26.6/63.35) live in areas not covered by the currently recommended high-risk surveillance. Of the 8.85 million women aged 40–59, it is estimated that 1%, 88,500, are eligible, and that up to 42% of these (37,167 women) are not receiving the appropriate surveillance. Although we do not have formal estimates of the absolute benefit of this surveillance, it is reasonable to assume that it is at least as effective as the moderate-risk surveillance. Thus, we estimate that up to 29 deaths per year would be prevented in the high-risk population if the guidelines for surveillance were followed.

With the recent publication of evidence that annual mammography screening in women aged 35–39 years at enhanced breast cancer risk appears equally effective at early detection^12^ compared to age 40–49,^8^ NICE is likely to evaluate this at the next update.^6^ However, one would have little confidence that any recommendation to offer screening aged 35–39 years would be fully implemented, despite the fact that women from regions covering a population of around 14.5 million appear to already get this (Table 1).

In the USA in 2007, the American Cancer Society (ACS) published recommendations for annual breast MRI along with annual mammography screening for women who have ≥20% lifetime risk for developing breast cancer incorporating both the majority of the UK moderate risk and all of the high-risk group.^15^ A survey in 2012 showed low access to MRI, although, understandably this was higher amongst those with health insurance.^16^ Overall there is relatively little published on adherence to breast screening guidelines internationally. That may be because it is difficult to audit or that it has not been explored. The Clinical Commissioning Groups (CGGs) look to save money and therefore are not willing to adopt all NICE guidelines. Not implementing this guidance would save on not having to provide funding for family history clinics for women to be assessed for BC risk. While CGGs appear to be able to ignore guidance, there are precedents to suggest that they could be successfully sued for not implementing NICE guidance without good reason (https://www.nice.org.uk/news/article/court-warns-ccg-over-disagreeing-with-nice-guidance). If CGGs were forced to adopt NICE guidelines this would reduce inequalities of access. Alternatively, all familial breast screening could be incorporated into the NHSBSP, which would also lead to more uniform access. Finally, it is important to educate and inform all women to have a breast cancer risk assessment by age 40 years, either through their GP or by developing tailored online tools to enable a self-assessment.

## Conclusion

We have shown that there has been only partial compliance with NICE approved mammographic screening surveillance in women at familial risk with less than half of England is now fully compliant. This has created a postcode lottery where women in one part of the UK get access to surveillance that their sister at the same risk cannot. It is a concern that even in a fully funded public health system such as the UK that major inequity in provision occurs even with the highest levels of evidence provided by NICE guidelines.

## Supplementary information

Supplementary Table

## Data Availability

All data are contained within the manuscript.
