# Supplementary material for: Sporadic implementation of UK familial mammographic surveillance guidelines 15 years after original publication
Source: Br J Cancer. 2019 Nov 25;122(3):329–32. doi: 10.1038/s41416-019-0631-2 (PMC7000386; doi:10.1038/s41416-019-0631-2)
Supplement: Supplementary file 1 — Supplementary Table [file 41416_2019_631_MOESM1_ESM.docx]

**Supplementary table**

1. What is your region?:
2. What population does your region serve?
3. Is moderate risk annual mammography screening aged 40-49 years available in your region? Yes, partially, No
4. Is high risk annual screening aged 40-59 in women who do not qualify for the NHSBSP very high risk screening available? Yes, partially, No
5. Is mammography screening under 40 years available outside NHSBSP? Y/N
6. Can you identify locations in your region where screening is NOT available:
